# Differentially Methylated Genes in Saliva are linked to Childhood Stress

**DOI:** 10.1038/s41598-018-29107-0

**Published:** 2018-07-17

**Authors:** Ligia A. Papale, Leslie J. Seltzer, Andy Madrid, Seth D. Pollak, Reid S. Alisch

**Affiliations:** 1 Department of Psychiatry, University of Wisconsin, Madison, USA; 2 Waisman Center, University of Wisconsin, Madison, USA; 3 Neuroscience training program, University of Wisconsin, Madison, USA; 4 Department of Psychology, University of Wisconsin, Madison, USA

## Abstract

Chronic and severe stress exposure in early childhood is associated with the development of psychiatric disorders. Yet, the molecular mechanisms underlying this relationship remain poorly understood. Here, we profile molecular marks (DNA methylation and gene expression) throughout the human genome to determine the associations between childhood stress exposure and gene regulation. To do so, we collected saliva tissue from prepubertal girls (mean age 10.9 ± 1.26 years) who had experienced different levels of childhood adversity, ranging from mild to severe. We found 122 differentially methylated genes (FDR *P*-value < 0.05) associated with high childhood stress exposures that affect brain development. Of these differentially methylated genes, 12 also differed in gene expression. To further investigate the potential effects of stress exposure on gene regulation, we examined the DNA sequences flanking all the differentially methylated loci. This analysis revealed enrichment of known binding sites for transcription factors, suggesting that DNA methylation may regulate gene expression by mediating transcription factor binding on these genes. Together, these findings indicate a possible neuromolecular mechanism linking children’s social experiences with risk for anxiety and depressive disorders.

## Introduction

Individuals who experience severe early life stress, such as physical abuse or neglect, are at heightened risk for a myriad of mental and physical health problems, including the development of emotional regulatory problems such as mood, anxiety, or aggressive disorders^1^. For these reasons, the impact of stress on young children’s biobehavioral development represents a major public health concern. Emerging research indicates that social environments can produce changes in gene expression. In this manner, life experiences can effectively “turn on” and “turn off” genes, leading to cascades of downstream changes in biology and behavior. Here, we examine environmental influences on gene expression that may reveal critical molecular mechanisms linking extreme childhood stress with the development of a host of health problems.

Epigenetic modifications, such as DNA methylation, are environmentally sensitive components in the regulation of gene expression. Therefore, this experience-dependent silencing or expression of genes, without changes in DNA sequence itself, may help account for behavior changes that have been observed in individuals who have endured early life stress. DNA methylation is a covalent modification that can occur on the DNA base cytosine when it is located next to guanine in the CpG dinucleotide^2^. This modification is less common in CpG-rich regions, known as CpG islands, which are located in the promoter region of many genes. Studies of adult post-mortem brain tissue support a role for DNA methylation in the development of emotion regulatory problems^3–8^. Our recent studies in young monkeys, as well as studies in humans, identified differentially methylated genes that are implicated as risk factors for anxiety and depressive disorders^9,10^.

A growing body of literature supports the hypothesis that DNA methylation has an important role in the risk to develop behaviors associated with early life stress, using sample sizes that ranged from 10–56 participants^10–18^. For example, alterations in DNA methylation abundance at the promoter of the glucocorticoid receptor gene are associated with abuse during childhood^19^, and disruptions in DNA methylation on the serotonin transporter gene functions as a biomarker of life adversity in humans^20^. These studies support a role for DNA methylation in the response to an early life adversity and warrant a deeper investigation at the genome-level.

Most of the extant research with humans has targeted single, specific genes, such as the glucocorticoid receptor. But given the complexity of human behavior, it is likely that many genes and gene regulatory sites (*e.g*., CpG dinucleotides) need to be considered to understand the link between stress and behavioral pathologies. Therefore we examined genome-wide alterations among stress-exposed children across nearly half a million sites throughout the human genome. We then tested which changes in DNA methylation were related to changes in gene expression, and whether the regulation of expression in these genes may be mediated by altered transcription factor binding.

## Method

### Participants

Twenty-two girls between 9 and 12 years of age (mean = 10.9 years, sd – 1 year, 2 months) participated in this study. Half of the children experienced normative levels of child stress exposure (average age = 11.23, SD = 1.34 years) and the other half had experienced extremely high levels of stress exposure (average age = 10.61, SD = 1.14 years). Children were recruited from a previous study about stress and hormone regulation^21^. Approximately half of the participants self identify their race to be Caucasian (13/22), and the number of participants from each race was not statistically different in each group. For gene expression analysis, children provided additional salivary samples. Four participants could not provide these samples, reducing the sample size for expression analysis to 18 (10 control, 8 high stress). All parents and participants gave informed consent/assent for the study and the University of Wisconsin–Madison Institutional Review Boards approved all procedures; all methods were carried out in accordance with the approved guidelines.

### Assessment of Childhood Stress Exposure

The Youth Life Stress Interview (for use with children) was derived from the UCLA Life Stress Interview, which was developed for use with adults. All items are open-ended interview questions administered separately to children and their parents. The interview is scored by an independent team of trained investigators. The interviewer does not participate in the scoring, so raters were unaware of the participant’s identity and childhood stress status. Children and their parents each separately completed portions of the Youth Life Stress Interview (YLSI; Rudolph *et al*., 2000) to elicit information about the adolescent’s life history. Graduate-level research staff administered this semi-structured interview after extensive training from Dr. Rudolph. A panel of trained raters then rated the extent of behavioral problems using a 5-point scale. Higher scores reflected more severe stress exposure. This measure demonstrates high reliability (average intraclass correlation = 0.96)^22^. In addition to the LSI, noted above, children completed the Children’s Depression Inventory^23^, the Revised Children’s Manifest Anxiety Scale^24^, the Child Behavior Checklist (CBCL)^25^.

### DNA Extraction and Methylation Detection

Saliva samples were collected using simple passive drool and Oragene kits for DNA methylation analysis (DNA Genotek, Canada). DNA was extracted following the manufacturers protocol. Genomic DNA samples were resolved on a 1% agarose gel, to verify the DNA was of high molecular weight, and quantified using Qubit (Qiagen, USA). DNA methylation levels were determined using the HumanMethylation450 array and beta values were obtained using R package minfi^26^. Beta values were background and control normalized, followed by subset-quantile within array normalization. CpGs were removed from analysis if they measured methylation at a SNP, contained a SNP within the probe, were known to be cross-reactive, or had at most one sample with a detection *P*-value > 0.01.

### Identification of Differentially Methylated Loci

Beta values were converted to M-value and R package limma^27^ was used to identify differentially methylated loci with a model using lifetime including score as a continuous variable and adjusting for age and beadchip. CpGs were ordered by chromosome and position, and P-values from limma were transformed to z-scores. A Hidden Markov Model was used to adjust for local index of significance (aLIS) using R package NHMMfdr^28^ with all parameters set to default. This model was employed for these studies for two reasons: 1) because mean methylation levels are strongly correlated across the genome and statistical power can be increased by ‘borrowing’ strength across adjacent measurements and 2) because the family-wise error rate is far too conservative when examining methylation data; instead, determining false discovery rates is a more appropriate way to analyze these data. Finally, CpGs were considered differentially methylated if the aLIS *P*-value was <0.05 (*N* = 550 differentially methylated loci [DMLs]). A Pearson’s correlation was identified for each DML. All methylation data was subjected to a surrogate variable analysis (R package *sva*) and zero surrogate variables were found in the data, suggesting that variables used in our model (*e.g*., LSI score, age) accounted for much of the observed variance, while latent confounders (*e.g*., cell type/count and ethnicity) were not present/sources of noise. Notably, all probes containing a single nucleotide polymorphism (SNP) were removed from the analysis. In addition, CpG IDs from all significant probes were placed into the mQTL database (http://www.mqtldb.org/search.htm), which utilized the MatrixEQTL database for association between SNPs and CpG ID. The developmental time point was set to “Childhood” and the distance was set to 200 base pairs.

### RNA Extraction, Sequencing, and Differential Analysis

Saliva samples were collected using simple passive drool and Oragene kits for RNA analysis (DNA Genotek, Canada). RNA was extracted following the manufacturers protocol. Total RNA for each sample was quantified using a bioanalyzer. Sequence libraries were prepared following the manufacturer’s guidelines (Illumina). In brief, first strand cDNA was end-repaired and ligated to unique adapters to generate sequence libraries. Library quality and quantity was determined on a bioanalyzer before sequencing on the Illumina HiSeq.2500 for 100-cycle paired-end sequencing. To identify differential whole-gene and/or isoform expression, FASTQ files from each sample were aligned to the human genome (hg19 assembly) using the GENCODE Human release 19 reference genome annotation. RSEM^29^ which utilized Bowtie (v0.12.7), was used to align reads to the genome, calculate read counts, and generate two separate data matrices, one for whole-gene read counts and one for isoform read counts. Read counts were log-transformed and quantile normalized using R package *preprocessCore*^30^. Differential expression was identified using R package *limma* and whole-genes/isoforms were considered significantly differentially expressed if the achieved *P*-value was <0.05 (*N* = 1,405 unique genes).

### Gene Ontological Analysis

Genes associated with DMLs were investigated for gene ontological enrichment of biological processes using R package clusterProfiler. The gene universe was used as background for enrichment (*N* = 21,232). A P-value of <0.05 cutoff was used on gene ontological terms with the addition of a fold-enrichment cutoff of >1.5 relative to the background. Gene ontologies were conducted using similar methods for differentially expressed genes.

### Transcription Factor Motif Discovery

The DNA sequences flanking DMLs (+/−250 bp) were used to identify enriched motifs using the DREME suite^31^. An *E*-value cutoff <10e-5 was used to identify motifs and putative binding factors were predicted using SpaMo directly from the DREME suite software package.

### Statistical analysis for molecular testing

Permutation testing was conducted to identify any significant over- or under-representation of DMLs at standard genic structures, in relation to CpG islands, and across chromosomes. The number of times that DMLs fell within each genomic structure was tallied and termed the “actual number” for each genomic structure. Notably, as some CpGs are associated with multiple genomic structures, each structure was used in permutation testing for each CpG. Next, genomic structures from all tested CpGs were obtained (*N* = 804,173) and the same number of structures as those from the DMLs were randomly selected, tallied, and termed the “permutated number” for each genomic structure. This calculation was generated 10e4 times. Each time the permutated number exceeded that of the actual number was tallied and divided by 10e4 to achieve the permutated *P*-value for each genomic structure. Similar methods were used for permutation testing of CpGs in relation to CpG islands. For permutation testing of chromosomes, similar methods were used, however, to correct for multiple testing (*i.e*., 22 autosomes and the X chromosome), proportions of DMLs that fell on each chromosome were calculated and the “actual proportion” of each chromosome was compared with the “permutated proportions” of any chromosome for each permutation. Separate permutations were utilized for all DMLs, positively-correlated DMLs, and negatively-correlated DMLs.

To identify significant enrichment for neuronal/immunological-related terms, a Pearson’s chi-square test with Yates’ continuity correction was conducted in R using a published list of neuronal/immunological-related gene ontological terms (*N* = 3,071)^32^.

Relations to known stress-related genes: A chi-square test was used to compare DML-associated genes (*N* = 122), differentially expressed genes (*N* = 1,405), and genes tested in the gene universe that are known stress-related genes extracted from the GeneCards database using the following terms: anxiety; bipolar; fear; depression; major depressive disorder; posttraumatic stress disorder; psychosis; schizophrenia; stress; and trauma (*N* = 4,286 (DNA methylation analysis); *N* = 3,070 (gene expression analysis)). Notably, the gene universe used for the chi-square test consisted of all the genes associated with the tested CpGs, that were tested for gene expression after filtration, or all genes tested in both analyses for the overlap between the two datasets (*N* = 20,741 (DNA methylation analysis); *N* = 13,585 (gene expression analysis); *N* = 23,407 (overlap)).

## Results

### Behavioral Phenotypes

As expected, children with high levels of life stress exposure were experiencing more behavioral problems (assessed via the Child Behavior Checklist), R^2^ = 0.28; *P*-value < 0.006. The specific symptoms expressed by children are presented in Table 1.

**Table 1:** Summary of behavioral phenotypes in children exposed to high stress (as assessed via the Child Behavior Checklist), at the time of saliva collection.

	R^2^	*P*-value
Incidence of Externalizing Symptoms	0.28	<0.006
Difficulties with Social Interactions	0.51	<0.0001
General Social Competence	0.39	<0.001
Reaction to Upsetting Thoughts	0.28	<0.007
Rates of Internalizing Symptoms	0.28	<0.007
Engagement in Extracurricular Activities	−0.56	<0.0001
Clinical Diagnosis of Anxiety Disorder	0.31	<0.004
Overall Problems with Behavior	0.28	<0.006

### Genes Showing Differential Methylation

**Figure 1:**
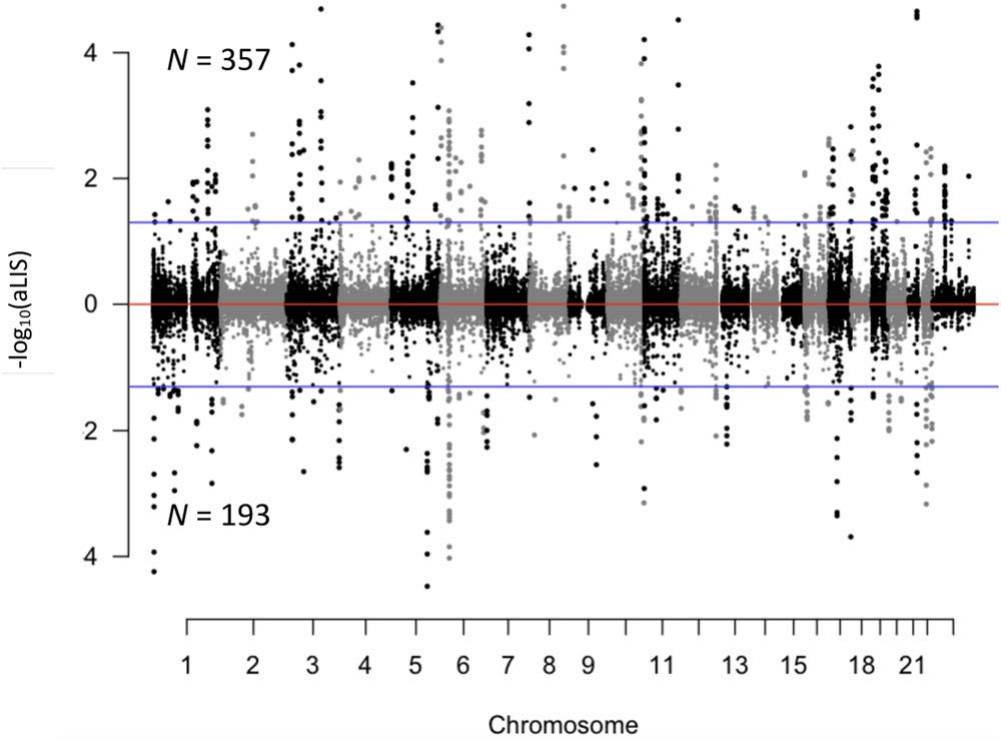
Characterization of DMLs across the human genome. Modified manhattan plot of CpG dinucleotides examined in this study (dots) reveals differentially methylated loci (DMLs) to be distributed across the entire genome. Positively and negatively correlated DMLs are displayed with the −log10 of the aLIS *P*-value. While all loci examined alternate between black and gray dots, to indicate each chromosome, significant DMLs are displayed outside of the dashed lines (aLIS *P*-value < 0.2).

To determine the extent to which early childhood stress exposure was associated with DNA methylation levels, we analyzed each CpG dinucleotide using a regression model and the lifetime stress (LSI) score as the explanatory variable. This analysis identified 550 genomic positions (loci) that were differentially methylated based on the children’s level of stress exposure. These differentially methylated loci (DMLs) were distributed across all chromosomes and had some preferences for specific genomic and gene structures (FDR *P*-value < 0.05; Fig. 1; Supplementary Fig. 1; Dataset 1). The relationships between stress exposure and DNA methylation included both positive (positive-DMLs; *N* = 357) and negative correlations (negative-DMLs; *N* = 193). To test if underlying genetic polymorphisms might explain these stress-associated changes in DNA methylation, we cross-reference of all the single nucleotide polymorphism (SNPs) in the mQTL database with the 550 differentially methylated sites and found that the majority (*N* = 456/550) of DMLs were located more than 200 base pairs from any reported SNP. Together, these data suggest early childhood stress exposure results in stable changes in DNA methylation.

**Figure 2:**
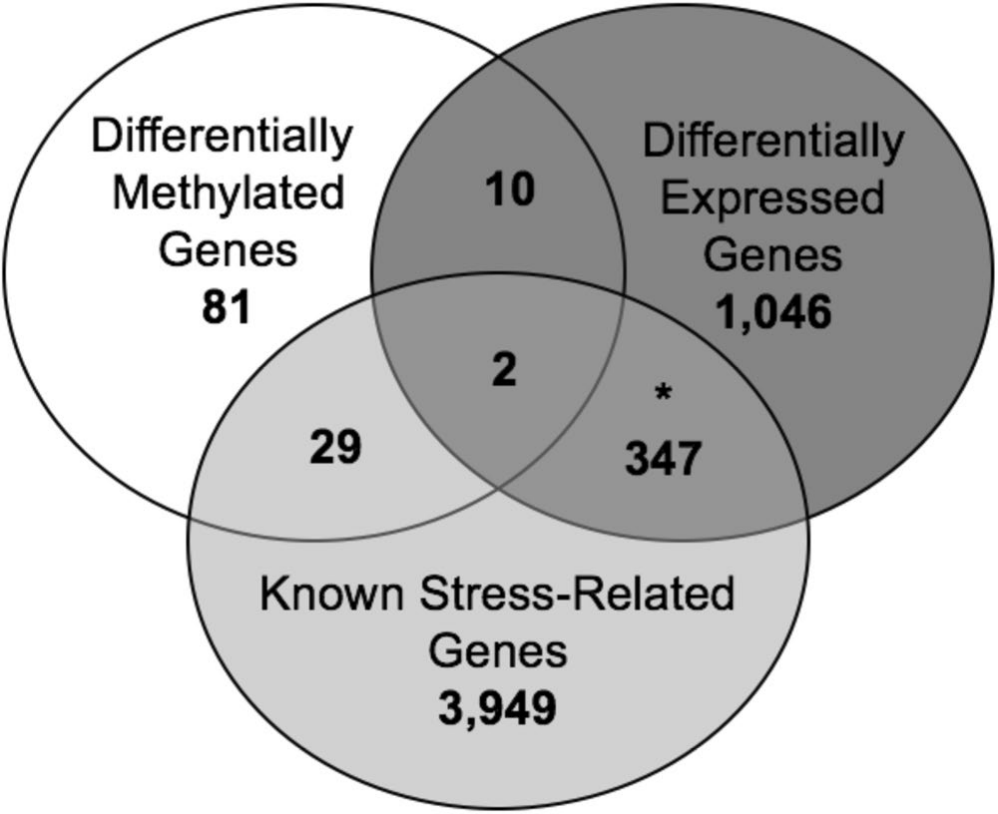
Overlap of differentially methylated and expressed genes. Venn diagram of the overlap between differentially methylated genes (*N* = 122) and differentially expressed genes (*N* = 1,405) with known stress-related genes tested within the gene universe (yellow; *N* = 4,327). An asterisk indicates that the overlap is significant (Chi-square *P*-value < 0.05).

Classification of the 550 stress-associated DMLs with the nearest gene revealed differential methylation associated with early stress exposure on 122 different genes, as some genes carried more than one DML. Of these, 84 genes were positively and 50 genes were negatively correlated with early stress exposure; 12 genes contained both positive- and negative-DMLs at different positions (loci) in the genes. The relationships among biological pathways for these 122 genes contained a significant enrichment of biologically relevant terms, including the regulation of neurotransmitter levels and neurotransmitter secretion (*χ*^2^ *P-*value < 0.05; Dataset 1). In addition, we compared the DML-associated genes to a list of known stress-related genes using the GeneCards database and found that 31 of the 122 genes had well-documented roles in response to stress (Fig. 2; Dataset 1). Together, these data suggest that stress-associated changes in DNA methylation target biologically relevant genes throughout the genome.

### DNA Methylation Differences Associated with Gene Expression

To determine if the stress-associated DNA methylation differences that we found have functional significance, we examined the extent to which these methylation changes were related to altered gene expression levels, using RNA sequencing (RNAseq). Comparison of transcript levels, using a linear regression model and the lifetime stress (LSI) score, revealed 1,405 unique genes that were differentially expressed at the whole-gene and/or isoform level (*P*-value < 0.05; Fig. 2; Dataset 2). Notably, 349 of the 1,405 differentially expressed genes previously have been implicated in stress regulation, suggesting that the remainder genes (*N* = 1056) have a novel role following early life stress exposure (*χ*^2^ *P*-value < 0.05; GeneCard database; Dataset 2). Similar to the differentially methylated genes, the differentially expressed genes also had a significant enrichment of biologically relevant relationships among them, which included the terms neuron projection regeneration and axon regeneration (*χ*^2^ *P*-value < 0.05; Dataset 2).

While genes that are both differentially expressed and differentially methylated represent an independent validation of stress-induced molecular changes, these associations also reveal candidate sites of functional DNA methylation, which may have a direct role in gene regulation. Overlaying the differentially methylated genes with the differentially expressed genes revealed 12 genes that harbor potentially functional DNA methylation changes linked to early-life stress exposure (Dataset 1). Two of these genes (*FHL3* and *NPC2*) are biologically relevant, with known roles in response to stress (see discussion).

**Figure 3:**
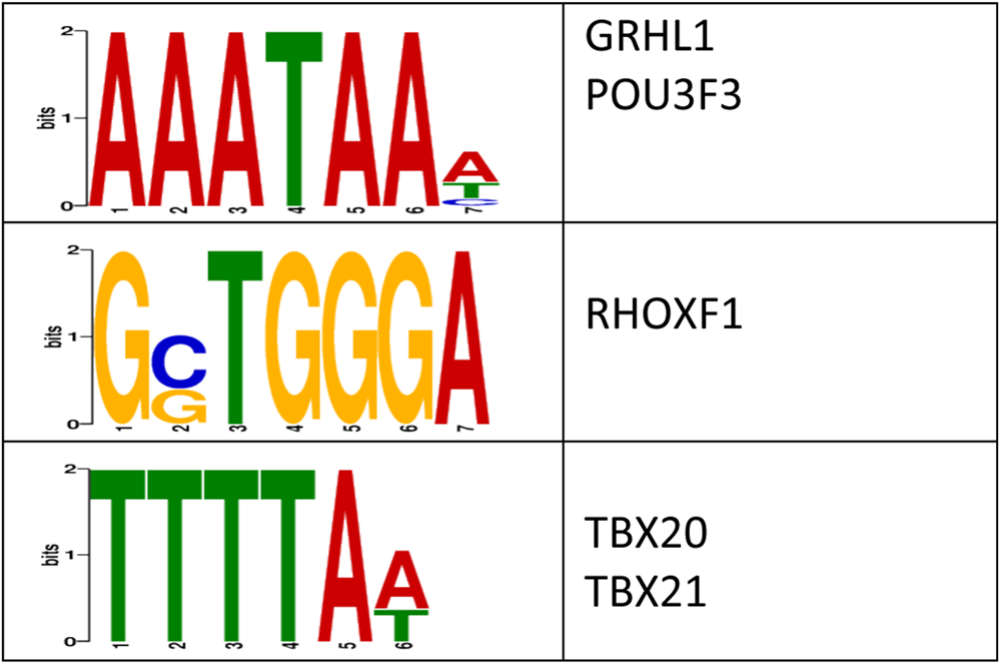
Characterization of the potential role(s) of DMLs on gene expression. The logo plots are shown for the enriched DNA sequence motifs that were predicted by the DREME suite using sequences from all DMLs (*E*-value < 10e-3). The transcription factors predicted to bind to each motif is shown to the right of each logo plot.

We next examined whether the stress-related changes in DNA methylation levels were enriched with transcription factor (TF) binding sites^31^. This analysis revealed a significant enrichment of three sequences that preferentially bind to five transcription factors, some of which have links to stress-related processes (*e.g., TBX21*; Fig. 3)^33^. Together, these data suggest that differential methylation levels may modulate transcription factor binding, which in turn alters gene expression, following exposure to early-life stress.

## Discussion

Here we present evidence that extremely high early life stress exposure in children is associated with changes in saliva DNA methylation levels, and that some these methylation changes are linked to gene expression. Of the 122 differentially methylated genes we detected, 31 have been implicated in stress-related functions, including genes involved in neurotransmission (*e.g*., social-related neuropeptides: *OXT* and a serotonin receptor: *HTR3A*)^34–39^. The other ninety-one genes not previously linked to stress may represent novel stress-sensitive genes involved in development. Further study of how and why childhood stress exposure alters the distribution of DNA methylation on these genes can improve our understanding of stress response and interventions aimed at recovery.

The overlay of methylome and transcriptome data found genes with potentially functional differential methylation, providing evidence that changes in DNA methylation regulates stress-induced gene expression. We identified 52 potentially functional differentially methylated sites that are associated with twelve differentially expressed genes (some genes had more than one differentially methylated sites). Two of these genes were previously linked to stress, *FHL3* and *NPC2*. *FHL3* (Four and a half LIM domain 3) proteins are members of the LIM protein superfamily that can function as co-activators of CREM/CREB transcription factors and the androgen receptor^40–42^. While the exact role of *FHL3* is not well understood, the data reported here warrants further investigation into its role in response to stress. Mutations in *NPC2* (Niemann-Pick type C2) lead to cholesterol accumulation in late endosomes and impaired cellular cholesterol homeostasis. Over recent years, it has become apparent that increased cholesterol in brain mitochondria can affect mitochondrial function, leading to more sensitivity to oxidative stress and decreased rates of ATP synthesis under certain conditions^43–45^. Thus, these data are consistent with the view that cerebral energy metabolism is part of social behavior, and points to mitochondrial function as a component of mood disorders^46^.

Transcription factors function to activate or repress gene expression upon binding to genes. The majority (7/12) of the differentially methylation sites that were correlated to altered gene expression contained DNA sequences that facilitate transcription factor binding. These data suggest that the functional role for stress-induced changes in DNA methylation might involve the modulation of transcription factor binding/function. It is notable that the transcription factor binding sites identified in this study recruit transcription factors implicated in stress-related disorders^33^. Together, these data support previous studies^47–49^ and suggest that DNA methylation may modulate transcription factor binding on developmentally important genes in the brain. It will be important that future studies verify this link using *in vitro* approaches.

A limitation to this study is the examination of a peripheral tissue in brain-related disorders. However, recent reports have shown correlations between DNA methylation levels in peripheral tissue and brain, including saliva^50–52^. Thus, DNA methylation content in peripheral tissues may be relevant, perhaps as a biomarker that could be used for diagnosis of early-life stress. Finding that ninety-four DMLs are within 200 base pairs of a reported mQTL suggests that at least some of these changes in DNA methylation are driven by genotype. Future studies may consider functional approaches to determine the genetic influence of these SNPs in the context of early life stress. It should be noted that while independently validated genes (*i.e*., both differentially methylated and expressed) represent top candidates of stress-induced molecular changes, the remaining differentially methylation genes are of interest and only await formal validation. Since epigenetic marks can be cell-type specific, it will be important for future studies to characterize the developmental timing of behavior-related effects on the epigenome and transcriptome in a biologically relevant cell-type (*e.g*., GABAergic interneurons) to improve early therapeutic interventions. Future studies might incorporate longitudinal designs to examine the origins of these and other molecular changes by profiling earlier developmental time-points.

The molecular factors underlying children’s vulnerability to environmental stressors have, thus far, been elusive. Epigenetic mechanisms are emerging as an important window into how early life experiences can affect developmental biology. A clearer understanding of the molecular mechanisms regulating the expression of the genes reported here may index modifiable molecular substrates that could ultimately be targeted to prevent the onset of some forms of psychiatric-related disorders.

### Data Access

We have submitted the methylation and expression data from this study to the Gene Expression Omnibus (GEO), which can be found under the Gene Series: GSE112314.

## Electronic supplementary material

Supplementary Figure

Dataset 1

Dataset 2
