# Supplementary material for: Differentially Methylated Genes in Saliva are linked to Childhood Stress
Source: Sci Rep. 2018 Jul 17;8:10785. doi: 10.1038/s41598-018-29107-0 (PMC6050255; doi:10.1038/s41598-018-29107-0)
Supplement: Supplementary file 1 — Supplementary Figure [file 41598_2018_29107_MOESM1_ESM.pdf]

Differentially Methylated Genes in Saliva are linked to Childhood Stress

Ligia A. Papale[1], Leslie J. Seltzer[2], Andy Madrid[1,3],

Seth D. Pollak[2,4], and Reid S. Alisch[1]

University of Wisconsin- Madison

Author Affiliations:

[1] Department of Psychiatry, University of Wisconsin – Madison

[2] Waisman Center, University of Wisconsin – Madison

[3] Neuroscience training program, University of Wisconsin – Madison

[4] Department of Psychology, University of Wisconsin – Madison

[*]The first two authors contributed equally to this work.

[*]Correspondence to:

Seth Pollak
Waisman Center, 399
University Wisconsin – Madison
1500 Highland Ave
Madison, WI 53705-2280
E-mail: spollak@wisc.edu
Telephone: (608) 890-2525
Fax: (608) 890-2424

Reid S. Alisch
Department of Psychiatry
University Wisconsin – Madison
6001 Research Park Blvd.
Madison, WI 53719
E-mail: alisch@wisc.edu
Phone: (608) 262-8430
Fax: (608) 263-9340

Supplementary Figure 1: Distribution of DMLs across standard genomic structures. The percent distribution (y-axis) of all CpGs tested (black), all DMLs (white), hyper-DMLs (grey), and hypo-DMLs (striped) across each structure (x-axis) is shown. Significant over- and under-representation of DMLs, determined by permutation testing, are indicated (*) for each genomic structure (*P*-value < 0.05). (A) Distribution of DMLs across genic structures. The genic structures include the following: 1,500bp upstream of the transcription start site (TSS1500); 200bp upstream of the transcription start site (TSS200); 5' untranslated region (UTR); 1st exon; gene body; 3' UTR; and intergenic regions. (B) Distribution of DMLs in relation to CpG islands. Structures related to CpG-islands include the following: North Shelf (2-4kb from island), North Shore (0-2Kb from island), CpG Island, South Shore, South Shelf, Open Sea (>4kb away from island). (C) Distribution of DMLs across the human chromosomes.

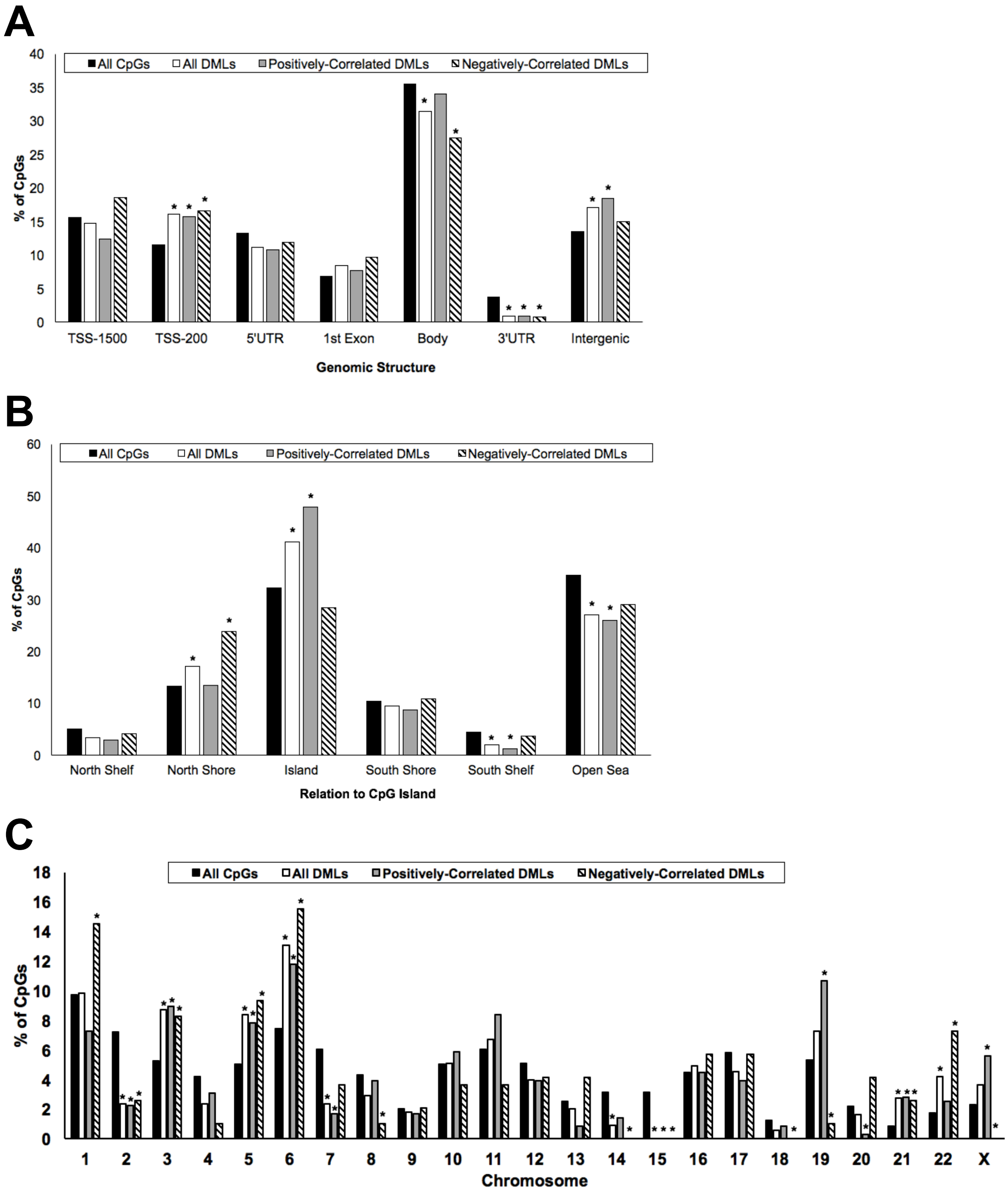
A
All CpGs
All DMLs
Positively-Correlated DMLs
Negatively-Correlated DMLs
% of CpGs
0
5
10
15
20
25
30
35
40
TSS-1500
TSS-200
5'UTR
1st Exon
Body
3'UTR
Intergenic
Genomic Structure
B
All CpGs
All DMLs
Positively-Correlated DMLs
Negatively-Correlated DMLs
% of CpGs
0
10
20
30
40
50
60
North Shelf
North Shore
Island
South Shore
South Shelf
Open Sea
Relation to CpG Island
C
All CpGs
All DMLs
Positively-Correlated DMLs
Negatively-Correlated DMLs
% of CpGs
0
2
4
6
8
10
12
14
16
18
1
2
3
4
5
6
7
8
9
10
11
12
13
14
15
16
17
18
19
20
21
22
X
Chromosome
